# Commentary: Prevalence and incidence of celiac disease in patients with rheumatoid arthritis: a case-control study based on the RECORD cohort

**DOI:** 10.3389/fmed.2025.1701521

**Published:** 2025-11-25

**Authors:** Garifallia Sakellariou, Annalisa Schiepatti, Anna Zanetti, Carlomaurizio Montecucco, Federico Biagi, Carlo Alberto Scirè

**Affiliations:** 1 Department of Internal Medicine and Therapeutics, Università di Pavia, Pavia, Italy; 2 General Medicine, Istituti Clinici Scientifici Maugeri IRCCS Pavia, Pavia, Italy; 3 Gastroenterology Unit, Istituti Clinici Scientifici Maugeri IRCCS, Pavia, Italy; 4 Società Italiana di Reumatologia Epidemiology Unit, Milan, Italy; 5 Division of Rheumatology, Fondazione IRCCS Policlinico San Matteo, Pavia, Italy; 6 School of Medicine, University of Milano Bicocca, Milan, Italy

**Keywords:** rheumatoid arthritis, coeliac disease, epidemiology, comorbidity, disability

## 1 Introduction

We read with interest the commentary by Bettacchioli et al. (1) on our article on the prevalence and incidence of coeliac disease (CD) in patients with rheumatoid arthritis (RA), based on administrative data of the Lombardy region, Italy (2). With our study as a start, the authors raised some interesting points of discussion regarding some open questions about the optimal testing strategy for CD in other autoimmune disorders. In fact, as an increased prevalence of CD has been shown in many autoimmune diseases (3), serological testing for CD is currently recommended only in type 1 diabetes and autoimmune thyroid disorders while the optimal strategy in other autoimmune conditions, including RA, has yet to be established (4, 5). There is paucity of data about the prevalence of CD in RA (2, 3, 6), therefore, this still represents a relatively unknown area, in which further evidence is required for a translation in clinical practice.

## 2 Administrative data for research in uncommon diseases

While both RA and CD do not fall under the definition of rare diseases, they still have a relatively low prevalence in the general population (7, 8), and this limits the assessment of the co-occurrence of these conditions in cohort studies. While we agree that an optimal diagnosis of CD would be biopsy-confirmed, and that detailed RA and CD clinical information would be valuable to study the interplay between these conditions, cohort studies could very hardly assess such an interaction. Even multicentric studies would not reach the necessary power, as suggested by the results of the ESPOIR cohort where only one in 700 RA patients had CD while on our administrative database of 70 061 RA patients, derived from a population of about 10 million, we found 171 CD. While ESPOIR enrolled a younger sample, with higher likelihood of new CD diagnoses, this might be less representative of a typical RA population, compared to our population-based sample, and the fact that ESPOIR is an historical cohort, enrolled between 2003 and 2005, might further limit generalizability (6). Although diagnoses in administrative databases are prone to uncertainty, we can safely exclude to have classified as CD patients with self-made diagnoses because in Italy the exemption code for CD, that we used to define the disease in our database, can be released only in secondary and tertiary gastroenterology centers based on the results of a duodenal biopsy suggestive of CD. The low probability of an over-estimation is also suggested by a lower prevalence of CD than expected in our population.

## 3 The impact of screening of CD in autoimmune diseases

While we found an increased prevalence of CD in female patients with RA, with an Odds Ratio of 2.15 (2), it is remarkable that that we were far from suggesting a universal screening of this group or of all patients with RA. To put our findings into context, the incidence rate ratio (IRR) of CD and type 1 diabetes is estimated between 8 and 9.9, but this is lower in autoimmune thyroiditis (2–2.9), although in this population universal screening is recommended. In the same study the IRR for CD in RA was between 1 and 1.9, but there were no subgroup analyses, and we cannot exclude that females with RA would have a prevalence approaching that of autoimmune thyroiditis (3).

The sustainability of disease testing is a central aspect, and Bettacchioli et al. estimated such costs in our population (1). Although the cost for screening only females was lower, the overall expense was significant, but this should be interpreted on the background of a general population of more than 10 million inhabitants. Moreover, a complete economic evaluation should also consider the impact of a diagnosis of CD, in terms of both increased and reduced costs. A recent systematic literature review estimated the burden of CD diagnosis, taking also into consideration the costs of endoscopy, showing a higher cost per diagnosis than that calculated for our cohort (9). It should also be taken into consideration that the scenario of CD diagnosis in adults is changing, and we cannot rule out that in the future some subsets of patients will be diagnosed without the necessity of a biopsy. This will not imply a relevant increase in costs, as the diagnosis will be based on symptoms and a high autoantibody titer (10).

## 4 Conclusions

The debate on the optimal testing strategy for CD is an open matter of discussion, and the degree of uncertainty is even higher in patients with rheumatic diseases, as older evidence was based on weak study design (11), while only more recently larger and solid studies have been presented (2, 3, 6). In the specific setting of RA, considering the typical features of these patients, the limited knowledge on CD in older patients constitutes a further difficulty. To our knowledge, so far, the only attempt to characterize RA patients eligible populations for screening was that of our study (2). Although we do not support universal screening in this subgroup, we recommend keeping a higher level of alertness in these patients, looking for symptoms compatible with CD. We strongly believe that further research will be needed in this area, trying to compromise between the detailed clinical information of the clinical databases and the power of administrative data.
